# Prevalence, characteristics, and associated factors of abnormal sensory nerve conduction study of sural nerve in patients with traumatic spinal cord injury: a cross-sectional study

**DOI:** 10.1038/s41393-025-01164-z

**Published:** 2025-12-23

**Authors:** Nutchaya Kantasena, Siam Tongprasert, Sintip Pattanakuhar

**Affiliations:** 1 Department of Rehabilitation Medicine, Faculty of Medicine, Chiang Mai University, Chiang Mai, Thailand; 2 Department of Rehabilitation Medicine, School of Medicine, University of Phayao, Phayao, Thailand; 3 Department of Biomedical Engineering, Faculty of Engineering, University of Strathclyde, Glasgow, UK

**Keywords:** Somatic system, Spinal cord diseases

## Abstract

**Study design:**

a cross-sectional study.

**Objective:**

To determine the prevalence and associated factors of abnormal sensory nerve conduction study of sural nerve and to describe the characteristics of sural sensory neuropathy in patients with spinal cord injury (SCI).

**Setting:**

Electrodiagnostic unit, Department of Rehabilitation Medicine, Faculty of Medicine, Chiang Mai University.

**Methods:**

Patients with any level of SCI who had no evidence of lower motor neuron lesion at the sacral level who visited the outpatient department, inpatient department, and urodynamic clinic at Maharaj Nakorn Chiang Mai Hospital between October 2023 and November 2024 were recruited. Nerve conduction studies (NCS) were performed following the American Association of Neuromuscular & Electrodiagnostic Medicine (AANEM) Guideline. The primary assessment was sural sensory NCS then the prevalence of sural neuropathy was calculated. Demographic and medical parameters were collected and analyzed to demonstrate the associations with sural neuropathy.

**Results:**

Among 95 participants, 23 were diagnosed with sural neuropathy, indicating a prevalence of 0.24 (95%CI: 0.16–0.34). Sural neuropathies observed in all participants were categorized into a type without evidence of compressive neuropathy. The independent associated factors of sural neuropathy were female, time since SCI longer than 10 years, cervical SCI, and history of pressure injury at the ischium.

**Conclusions:**

In people with SCI, the prevalence of sural neuropathy is 24%. Due to limitations in the study design and data collection for detecting neuropathy and risk factors, further longitudinal studies are needed to understand the neurophysiological deterioration following SCI and to confirm these findings.

## Introduction

Spinal cord injury (SCI) refers to damage to the spinal cord, mostly resulting from trauma. SCI can involve spinal tracts, spinal neurons, and possibly spinal nerve roots [1]. Therefore, it should lead to upper motor neuron lesions or preganglionic lower motor neuron lesions, which can be confirmed by a normal sensory nerve conduction study (NCS), part of electrodiagnostic testing, unless there is concurrent peripheral neuropathy [2].

Despite being counterintuitive, previous studies reported that patients with SCI had abnormal sural sensory NCS [3–7]. The prevalence was varied, ranging from 7 [6] to 75% [3]. These discrepancies might be attributed to the small sample sizes in these studies, with a maximum of 42 participants [7]. Therefore, the exact prevalence of sural neuropathy in patients with SCI has yet to be elucidated. Also, no study has been proposed on the associated factors of sural neuropathy in patients with SCI. [8]

The sural nerve is an anastomotic branch of the tibial and peroneal nerves. It is a pure sensory branch innervating the skin of the lateral lower leg and foot. Sural NCS was most commonly studied as a representative of sensory NCS, as the sural nerve is the most frequently involved nerve under the length-dependent pathophysiologic mechanism [9]. Although various investigation protocols exist, the American Association of Neuromuscular and Electrodiagnostic Medicine (AANEM) has proposed a standardized protocol for sural sensory NCS, which has been widely accepted [10].

These findings from previous studies suggest that SCI can lead to peripheral neuropathy involving the sural nerve, a phenomenon that is difficult to explain under the preganglionic lesion theory alone. The inconsistent results across these studies raise concerns about the reliability of electrodiagnostic studies in patients with traumatic SCI, particularly for diagnosing complications such as peripheral nerve entrapment, plexopathy, or polyneuropathy. Also, if factors associated with sural neuropathy could be elucidated, clinicians can use them as the criteria for screening and monitoring the abnormality of the peripheral nervous system.

However, there is no study demonstrating the exact prevalence of sural neuropathy in patients with SCI using a standardized NCS protocol and criteria for diagnosis, in a pre-calculated adequate sample size. Also, there is no study demonstrating the influencing factors of sural neuropathy in patients with SCI. Therefore, the primary objective of this study was to explore the prevalence of sural neuropathy in patients with SCI. The secondary objectives were: 1) to describe the characteristics of sural neuropathy and 2) to identify the independent associated factors of sural neuropathy following SCI.

## Methods

This study was designed as a cross-sectional study. The protocol of this study was approved by the local research ethics board, Faculty of Medicine, Chiang Mai University (Study number: REH-2566-0232). All parts of the study were conducted at the Electrodiagnostic unit, Department of Rehabilitation Medicine, Faculty of Medicine, Chiang Mai University, between October 2023 and November 2024. All methods were performed in accordance with the ethical guidelines and regulations. Written informed consent was obtained from all participants.

### Participants

The study population was recruited from the rehabilitation outpatient department, inpatient department, and urodynamic clinic at Maharaj Nakorn Chiang Mai Hospital between October 2023 and November 2024. The inclusion criteria were: 1) age between 20 and 70 years old; 2) being diagnosed with traumatic SCI at any time since injury, any level, and having American Spinal Injury Association (ASIA) impairment Scale (AIS) A-D, with no evidence of lower motor neuron lesion (LMNL) at sacral level. Evidence of LMNL at the sacral level included: 1) being diagnosed with cauda equina syndrome; 2) being diagnosed with conus medullaris syndrome; and 3) evidence of spinal shock syndrome. The exclusion criteria were: (1) a documented diagnosis of peripheral neuropathy in the medical records, including polyneuropathy; 2) history of taking or currently taking induced peripheral neuropathy (such as amiodarone, chloramphenicol, isoniazid, corticosteroids, hydralazine, pyridoxine) [11]; 3) being diagnosed with lower extremities peripheral nervous system injury, syringomyelia; 4) contraindicated to electrodiagnostic study. Withdrawal criteria were: 1) incomplete NCS study; 2) obtaining a history of lower motor neuron lesions or concomitant non-traumatic SCI after conducting NCS.

### Sample size calculation

The primary outcome of this study is the prevalence of sural neuropathy. Therefore, the sample size was calculated by using a formula for estimating a ratio parameter of an infinite population [12]. With a Z_α_ of 1.96, P (expected proportion) of 0.36 (according to the study of Kirshblum, et al. [3]), and d (acceptable error) of 0.1. Therefore, the required sample size of this study was 89. However, we invited 100 participants to compensate for potential withdrawal.

### Study protocol

After giving informed consent, participants were interviewed, and their medical records were reviewed to obtain demographic and medical history data. Capillary blood glucose was measured at the time of data collection, regardless of whether participants had fasted (random test). Random blood sugar testing was performed to rule out diabetes mellitus (DM). If the random blood sugar were over 200 mg/dL, the participant would not be included in the study. After that, the electrodiagnostic study was performed according to the protocol described in the following section. All tests (interviews, random blood sugar test, and electrodiagnostic study) were completed on the same day for outpatient participants and during the same admission visit for inpatient participants, not more than 7 days apart.

### Electrodiagnostic study protocol

The Dantec Keypoint (Medtronic) Workstation, Model 22022, manufactured in Denmark, was used as the electrodiagnostic machine throughout the study. The electrodiagnostic assessment would be performed only when the measured skin temperature at the lateral side of the lower legs was above 32 degrees Celsius.

**Fig. 1:**
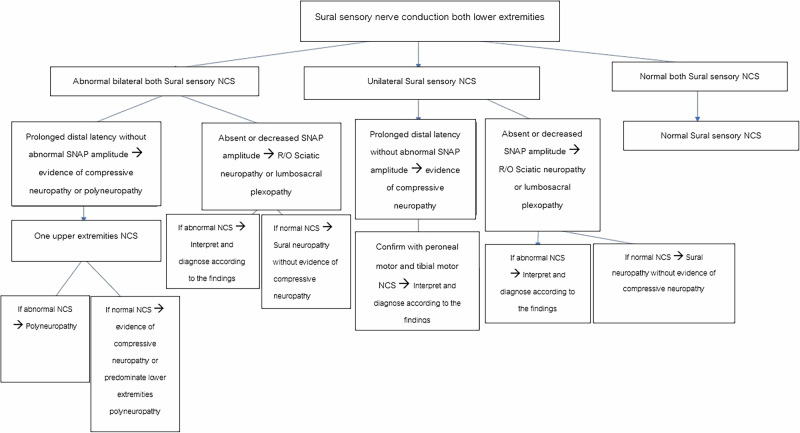
The flow of the electrodiagnostic study protocol. NCS nerve conduction study, SNAP sensory nerve action potential.

**Fig. 2:**
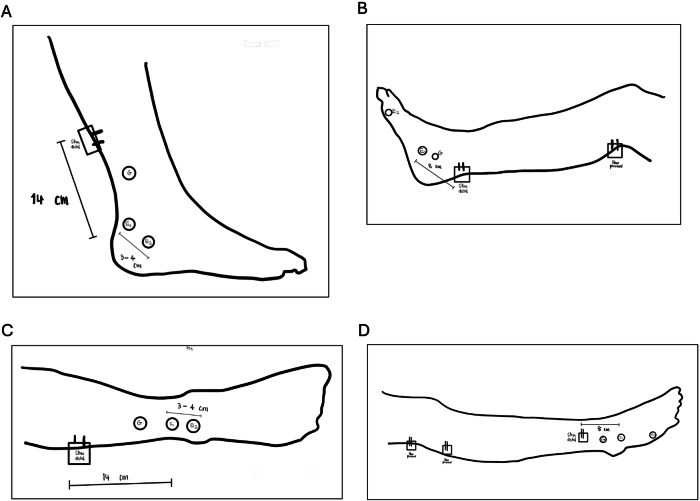
The methods for conducting nerve conduction studies. **A** Sural sensory nerve conduction study; **B** tibial motor nerve conduction study, recorded at AH muscle; **C** superficial peroneal sensory nerve conduction study; **D** peroneal motor nerve conduction study, recorded at EDB muscle. NCS nerve conduction study, SNCS sensory nerve conduction study, MNCS motor nerve conduction study, AH abductor hallucis, EDB extensor hallucis brevis.

Sural sensory nerve conduction studies (NCS) were performed according to the AANEM guidelines [10]. Since the primary focus of the study was the sural nerve, the sural sensory NCS was first performed. If the sural sensory NCS were normal, the NCS would finish. If the sural sensory NCS were abnormal, tibial and peroneal motor NCS, as well as superficial peroneal sensory NCS, would be conducted to rule out lumbosacral plexopathy or polyneuropathy. If there were suspicious evidence of polyneuropathy, such as prolonged distal latencies disproportionate to the degree of axonal loss, in all tested muscles, sensory and motor NCS of the median and ulnar nerves would be performed to confirm or exclude the diagnosis of polyneuropathy. Sural neuropathy was defined according to the AANEM Guideline [10]. The normal values for the sural SNCS are a peak distal latency ≤ 4.5 ms. and a SNAP amplitude ≥ 4 µV. The normal values for the other SNCS and MNCS are according to the AANEM guidelines, which have been presented elsewhere [10]. Figures 1 and 2 illustrates the flow of the electrodiagnostic study protocol and the methods for conducting NCS in this study, respectively.

### Variable determination

Continuous data were categorized as: 1) age was categorized as less than 50 years old vs more than or equal to 50 years old, according to the menopause age group, since it was hypothesized from the previous study that the higher prevalence of neuropathy in females was associated with hormone depletion from menopause [13]. Duration of SCI was categorized into 1) acute (less than 1 month); 2) post-acute (1 month to 1 year); 3) early chronic (1–5 years); 4) intermediate chronic (5–10 years); and 5) late chronic (more than 10 years). The level of the SCI was re-categorized into: 1) cervical SCI and 2) thoracolumbar SCI. Severity and completeness of SCI were categorized as: 1) complete SCI (AIS A) vs incomplete SCI (AIS B, C, D); motor complete SCI (AIS A and B) vs motor incomplete SCI (AIS C and D); and motor non-useful SCI (AIS A, B, C) and motor useful SCI (D).

### Statistical analysis

Statistical analysis was performed using STATA version 17.0. For descriptive statistics, the prevalence of abnormal sural sensory neuropathy was reported as a percentage with 95% confidence intervals (CI). For inferential statistics, bivariable associations between having sural neuropathy at the individual level and each influencing factor were analyzed using an independent t-test for normally distributed continuous parameters, a Mann-Whitney U test for non-normally distributed continuous parameters, and Fisher’s exact test with odds ratios and 95% confidence intervals for categorical parameters. Multivariable logistic regression was used to determine the independent factors associated with sural neuropathy at the individual level. Factors were included in the multivariable model if their p-value was less than 0.2, together with the important factors, including age, sex, level, and completeness of SCI, and time since injury, were included in the multivariable model as previously advised [14]. The modeling strategy was exploratory, i.e., including all factors without elimination. A p-value of less than 0.05 was considered statistically significant.

## Results

### Participant recruitment and characteristics

Between October 2023 and November 2024, 100 individuals with SCI were recruited for this study. Five participants were withdrawn from the study due to incomplete study (n = 1) and obtaining a history of lower motor neuron lesions or concomitant non-traumatic SCI after conducting NCS (n = 4). Therefore, 95 participants were included in the analysis. Notably, this sample size was still exceeding the calculated sample size of 89. Table 1 demonstrates the demographic and medical history data of the participants. According to the demographic parameters, the mean (SD) age was 45 (12) years and most of them were in the age group of less than 50 years old (n = 58, 61%). Most participants were male (n = 77, 61%). According to the SCI-relevant medical history, most of the participants had complete spinal cord injury (n = 59, 62%) and motor complete SCI (n = 76, 80%) whereas only 11 participants (12%) had useful motor functions. The majority of participants lived with SCI for more than 10 years (n = 39, 41%). The most common mechanism of SCI was a traffic accident (n = 59, 62%). The majority of participants underwent spinal surgery (n = 84, 90%). The most common site of pressure injury was the sacral area, which was present in 40 participants (42%). Notably, there was no participant currently using medications that can induce peripheral neuropathy, and no participant had been diagnosed with diabetes mellitus or peripheral neuropathy.

**Table 1:** Comparisons of characteristics between participants with and without sural neuropathy.

Parameters	Overall N = 95	Non-compressive sural neuropathy (bilateral/unilateral) n = 23 (24%)	No sural neuropathy n = 72 (76%)	p-value
Sex, n (%)				
Male	77 (81)	14 (61)	63 (87)	0.011*
Female	18 (19)	9 (39)	9 (13)	
Age (years), mean (SD)	45 (12)	49 (10)	44 (13)	0.074
Age group, n (%)				
Less than 50 years	58 (61)	11 (48)	47 (65)	0.149
50 years or more	37 (39)	12 (52)	25 (35)	
Level of injury, n (%)				
Cervical	38 (40)	10 (43)	28 (39)	0.808
Thoracolumbar	57 (60)	13 (57)	44 (61)	
ASIA Impairment Scale (AIS), n (%)				
AIS A	59 (62)	14 (61)	45 (62)	0.245
AIS B	17 (18)	7 (31)	10 (14)	
AIS C	8 (8)	1 (4)	7 (10)	
AIS D	11 (12)	1 (4)	10 (14)	
Complete SCI, n (%)	59 (62)	14 (61)	45 (63)	1.000
Motor complete SCI, n (%)	76 (80)	21 (91)	55 (76)	0.145
Motor useful SCI, n (%)	11 (12)	1 (4)	10 (14)	0.286
Time since injury group, n (%)				
Less than 1 month	3 (3)	0 (0)	3 (4)	0.012*
1 month - 1year	16 (17)	2 (9)	14 (19)	
1–5 years	27 (28)	3 (13)	24 (33)	
5–10 years	10 (11)	1 (4)	9 (13)	
More than 10 years	39 (41)	17 (74)	22 (31)	
SCI more than 10 years	39 (41)	17 (74)	22 (31)	<0.001*
Mechanism of injury, n (%)				
Traffic injury	59 (62)	13 (57)	46 (64)	0.684
Falling	27 (28)	8 (35)	19 (27)	
Penetrating	3 (3)	0 (0)	3 (4)	
Direct injury	2 (2)	1 (4)	1 (1)	
Others	4 (4)	1 (4)	3 (4)	
Vertebral surgery, n (%)	84 (90)	20 (87)	64 (91)	0.685
LE injury, n (%)	11 (12)	5 (22)	6 (8)	0.128
History of pressure injury (PI) at				
Sacrum/coccyx, n (%)	40 (42)	11 (48)	29 (40)	0.629
Ischium, Rt, n (%)	13 (14)	10 (43)	3 (4)	<0.001*
Ischium, Lt, n (%)	9 (9)	4 (17)	5 (7)	0.213
Ischium, either side, n (%)	19 (20)	11 (48)	8 (11)	<0.001*
Lower extremity, n (%)	12 (13)	5 (22)	7 (10)	0.155
DVT, n (%)	6 (6)	0 (0)	6 (8)	0.330
Spasticity medication, n (%)	49 (52)	12 (52)	37 (51)	1.000
Neuropathic pain medication, n (%)	43 (45)	8 (35)	35 (49)	0.337
Using any medication, n (%)	93 (98)	23 (100)	70 (97)	1.000
Underlying disease of diabetes, n (%)	0 (0)	0 (0)	0 (0)	1.000

*SD* standard deviation, *ASIA* American Spinal Injury Association, *SCI* spinal cord injury, *LE* lower extremity, *DVT* deep venous thrombosis.

Bolded, statistically significant at the level of *p* < 0.05 according to Fisher's exact test for categorical variables and the independent ttest for continuous variables.

### Prevalence and characteristics of sural neuropathy

Table 2 demonstrates the results of the prevalence and characteristics of sural neuropathy. Among 95 participants diagnosed with spinal cord injury, 23 participants had sural neuropathy on at least one side, indicating a prevalence of sural neuropathy of 0.24 (95%CI: 0.16–0.34) at an individual level. At the limb level, sural neuropathy was found in 36 of 190 tested limbs, yielding a prevalence of 0.19 (95% CI: 0.14–0.25). For the characteristics, all sural neuropathies were categorized as having no evidence of compressive neuropathy, as the sural sensory NCS demonstrated only axonopathy, either complete or partial, and no prolonged distal latency was found. However, severe nerve compression leading to complete axonopathy cannot be excluded due to the absence of distal stimulation. For instance, bilateral sural neuropathy was found in 12 participants (13% of all participants, 57% of participants with sural neuropathy). Among those with bilateral sural neuropathy, complete axonal loss sural neuropathy, indicated by the absence of SNAP, was found in 10 participants (83% of participants with sural neuropathy). Unilateral sural neuropathy was found in 11 participants (12% of all participants, 43% of participants with sural neuropathy). Rt and Lt complete axonal loss sural neuropathy was found in 18 and 13 participants (86 and 87% of participants with sural neuropathy on that side, respectively).

**Table 2:** Characteristics of sural neuropathy.

Characteristics	n (% of all participants)
Sural neuropathy, either side, any type	23 (24)
Sural neuropathy with evidence of compressive neuropathy	0 (0)
Sural neuropathy without evidence of compressive neuropathy	23 (24)
Bilateral sural neuropathy	12 (13)
Unilateral sural neuropathy	11 (12)
Rt sural neuropathy	21 (22)
Lt sural neuropathy	15 (16)
Characteristics	**n (% of participants with sural neuropathy)**
Absent SNAP of sural nerve, bilateral (complete axonal loss)	10 (83)
Absent SNAP of Rt sural nerve (complete axonal loss)	18 (86)
Absent SNAP of Lt sural nerve (complete axonal loss)	13 (87)

*SNAP* sensory nerve action potential.

### The influencing factors of sural neuropathy determined by bivariate analysis

Comparisons of characteristics between participants with and without sural neuropathy are demonstrated in Table 1. When compared with participants without sural neuropathy, those with sural neuropathy had significantly more females (39 vs 13%: p = 0.011, Fisher exact test), a higher percentage of participants who lived with SCI more than 10 years (74 vs 31%, p < 0.001, Fisher exact test), a higher percentage of participants who had a history of pressure injury on the right ischium (43 vs 4%, p < 0.001, Fisher exact test), and history of pressure injury on either side of the ischium (48 vs 11%, p < 0.001, Fisher exact test).

### The independent influencing factors of sural neuropathy determined by multivariable analysis

Since the factor of having a history of pressure injury on the right ischium and the factor of having a history of pressure injury on either side of the ischium represent a nearly similar issue, only the factor of having a history of pressure injury on either side of the ischium was included in the model to avoid the colliding effect. Also, motor complete SCI (p = 0.145) was selected over complete SCI (p = 1.000), as the former had a lower p-value. Therefore, the factors included in the final model were age group of 50 years or older, female sex, cervical level, motor complete SCI, time since injury of more than 10 years, history of pressure injury on either side of the ischium, and history of lower extremity injury. Results of the multivariable logistic regression analysis demonstrated that the independent associated factors of sural neuropathy were female sex (odds ratio = 6.088 [95% CI: 1.535–24.157], p = 0.010), cervical SCI (odds ratio = 5.217 [95% CI: 1.206–22.569], p = 0.027), and a history of pressure injury on either side of the ischium (odds ratio = 8.556 [95% CI: 1.898–38.560], p = 0.005) (Table 3). These results can be interpreted that, if other factors are held constant, being female, having cervical SCI, and having a history of pressure injury on either side of the ischium would increase the risk of having sural neuropathy by six, five, and approximately nine times, respectively.

**Table 3:** Independent factors associated with sural neuropathy in individuals with SCI from multivariable logistic regression analysis.

Independent variables	B	Exp (B) (Odds ratio)	95% CI of Odds ratio		p-value
			Lower	Upper	
Age more than 50 years	0.633	1.883	0.548	6.476	0.315
Sex, female	1.806	6.088	1.535	24.157	0.010*
Time since SCI more than 10 years	1.465	4.328	1.080	17.343	0.039
Cervical SCI	1.652	5.217	1.206	22.569	0.027*
Motor complete SCI	0.867	2.380	0.373	15.196	0.359
Concomitant lower extremity injury	0.825	2.282	0.509	10.239	0.281
History of having pressure injury at either side of ischium	2.147	8.556	1.898	38.560	0.005*

B, regression coefficient, *CI* confidence interval, *SCI* spinal cord injury.

Bolded, statistically significant at the level of *p* 0.05 according to the multivariable logistic regression analysis.

## Discussion

### The prevalence of sural neuropathy in patients with SCI

Results of this study confirm that sensory nerve abnormality is present in patients with SCI, with the largest sample size that has ever been used and following the standard guideline of AANEM. Notably, this study used sural sensory NCS as a representative of sensory neuropathy, consistent with the theory of length-dependent axonal degeneration, the most common characteristic of peripheral neuropathy [15].

The prevalence of sural neuropathy after SCI was 24% in this study. This prevalence was comparable with the previous study of Kirshblum, et al. [3], which demonstrated a prevalence of 36% [3]. However, it differed from those in previous studies, which demonstrated either higher [4, 5] or lower [6, 7]. The main explanation for this inconsistency was the difference in sample size, since all previous studies had participant numbers between 12 and 42, which may have led to alpha and beta errors due to inadequate sample size [16]. Other contributing factors include differences in the associated factors across studies, including sex, time since SCI, and history of pressure injury on either side of the ischium, which will be discussed later.

### Characteristics of sural neuropathy

All sural neuropathies presented in this study were diagnosed without evidence of compressive neuropathy, i.e., only decreased or absence of SNAP amplitude was found. However, the absence of SNAP amplitude may not guarantee that focal slowing, as a sign of compressive neuropathy, has not previously occurred before the condition of complete axonopathy we cross-sectionally found in this study. Therefore, we refrained from using the term “non-compressive neuropathy” and used the term “no electrophysiologic evidence of compressive neuropathy” instead.

In this study, there are two types of neuropathies. Half of them had bilateral neuropathy whereas the other half had unilateral neuropathy. Despite more common results from systemic conditions, bilateral sural neuropathy can also be caused by local injuries on both sides. On the other hand, unilateral sural neuropathy is more likely to result from local injury, including compressive neuropathy from unaware repetitive movements, causing neuropathic nerve injuries and direct injuries to the nerve from pressure injury [5]. This etiology was supported by our result, which demonstrated a significant positive association between having pressure injury on either side and sural neuropathy.

### Factors associated with sural neuropathy in patients with SCI

Female sex was a significant positive associated factor of sural neuropathy. Notably, the female proportion in this study was comparable to that in the SCI population in Thailand (72%) [17]. We first hypothesized that the effect of female sex on sural neuropathy should be associated with postmenopausal changes in hormones. Therefore, we have adjusted for age by categorizing it into two groups — under 50 years old and over 50 years old —based on knowledge of hormonal changes during this period [13] and controlled for it in our multivariable logistic regression model. However, after controlling for the age group factor, the female sex was still an independent positive associated factor of sural neuropathy. The mechanisms of a higher risk of sural neuropathy in females have yet to be explored.

The second independent positive associated factor of sural neuropathy was a pressure injury on either side of the ischium. We suspected that this finding may be due to the direct involvement of the pressure injuries. However, our post-hoc analysis matching on ipsilateral sural neuropathy and pressure injury demonstrated a significant positive association only on the right side, not on the left (p < 0.001 and p = 0.346, respectively, Fisher’s exact test). This inconsistency may be due to a relatively low prevalence of both sural neuropathy and pressure injury at the ischium on the right side, causing an underpowered statistical analysis. Also, we found that two of the twelve participants with a history of pressure injury on either side of the ischium had bilateral sural neuropathy, which could not be explained by the direct involvement of pressure injury. Further longitudinal studies should be conducted to prove the causation between sural neuropathy and pressure injury.

The multivariable logistic regression analysis demonstrated that cervical-level SCI was a significant positive associated factor of sural neuropathy after controlling for other factors. The mechanism of this association has yet to be explored. We hypothesized that cervical-level SCI may be associated with prolonged critical illness and undernutrition [18], then potentially cause a risk of developing malnutrition-associated neuropathy. However, further studies aiming to investigate this association should be conducted by assessing both nutritional status and electrophysiologic function simultaneously.

Results of this study demonstrated that time since injury was a significant positive factor associated with sural neuropathy. Living with SCI longer than 10 years may increase risks of malnutrition, pressure injury, chronic repetitive injury, and falls, together with an accelerated aging process [19]. These changes may be a contributing factor to the increasing prevalence of sural neuropathy according to age.

Other factors, including age group, injury to the lower extremities, use of spasticity medications, and use of neuropathic pain medication, were not significantly associated with sural neuropathy. These insignificant results may be due to the inadequate prevalence of both independent and dependent factors, resulting in insufficient power in the statistical tests. Further studies, especially those including more events of lower extremity injury, are needed to explore this association.

### Proposed mechanism of sural neuropathy following SCI

Although the presence of sural neuropathy is repeatedly confirmed, the underlying mechanisms cannot be determined in this study. We would categorize the underlying mechanisms of SCI-related sural neuropathy into intrinsic and extrinsic mechanisms. For the intrinsic mechanisms, i.e., those that occur without any concomitant peripheral neuropathy, Moon and colleagues (2020) proposed that it may be associated with transsynaptic degeneration of the dorsal root ganglion, proven to occur in motor neuropathy after SCI [20], and associated with decreased brain-derived neurotrophic factor and neurotrophins in motor neurons [21]. For extrinsic mechanisms, several causes of polyneuropathy were not thoroughly evaluated in this study or in previous studies, including vitamin B12 insufficiency, which occurs in 13.3% of SCI patients [22]. Further studies are needed to elucidate the responsible mechanisms of sensory neuropathy following SCI.

### Strengths and limitations

This study has some strengths. First, it has a sufficiently large sample size, which was calculated to confirm the adequacy of statistical power to detect the true prevalence of sural neuropathy. Also, sural neuropathy was diagnosed according to the guidelines provided by AANEM, which is standardized and should be valid. Another strength of this study is that all NCS were performed solely by the first author (N.K.), who has 2 years of experience in electrophysiologic testing, ensuring consistent NCS technique throughout the study.

This study has some limitations. First, since we primarily aimed to evaluate the prevalence of sural neuropathy, other NCS and electromyography were not entirely performed. Therefore, we cannot determine the exact location of the lesion, nor its characteristics and pathophysiology. Next, the participants would have a risk of compressive sural neuropathy if there were a demyelinating pattern in sural SNCS. However, this may underestimate the compressive pathology, as complete compression may result in an absent SNAP or conduction block without a demyelinating pattern on sural SNCS. For the evaluation of plexopathy, we performed NCS according to the protocol that requires abnormalities in other studies, such as the superficial peroneal SNCS, peroneal MNCS, and tibial motor NCS. But it cannot differentiate between symmetrical polyneuropathy and bilateral plexopathy.

Another limitation is that the exclusion criteria were solely based on medical records, specifically for peripheral neuropathy (including polyneuropathy). Therefore, undiagnosed causes of polyneuropathy, such as vitamin B12 deficiency, chronic inflammation/infection, systemic diseases, and intoxication, cannot be determined in participants; then this condition cannot be excluded. Additionally, using only a random blood glucose level (greater than 200 mg/dL) to exclude those with diabetes would be insensitive. More sensitive measures, such as fasting plasma glucose or HbA1c, should be required for more accurate exclusion in a future study. This limitation arises because some participants in this study were recruited after their normal annual follow-up session, so the definitive diagnostic criteria for diabetes cannot be applied to them (or else we would lose this group of patients).

Although our results showed that cervical-level spinal cord injury was associated with sural neuropathy, the level of functional dependence, including ambulatory status, wheelchair use, and level of physical activity, was not collected in our dataset, despite these factors potentially influencing the development of sural neuropathy. As our objective is to demonstrate the prevalence of sural neuropathy in patients with SCI, regardless of their chronicity and ambulatory status, our inclusion criteria included participants with varying chronicity and ambulatory statuses, resulting in heterogeneity within the study population. We attempted to adjust for this factor in our multivariable analysis using the “time since SCI more than 10 years” and “AIS” parameters, as it may partially (but not entirely, due to the inadequate size of each subgroup) reflect chronicity and ambulatory status. Further studies should address these crucial factors.

Next, although the sample size was calculated, it was based on the primary outcome of prevalence. It may not be adequate to detect the associations between sural neuropathy and influencing factors. Additionally, the cross-sectional design prevents causal inference of the results; therefore, further longitudinal cohort studies are needed.

### Clinical and research implementation

As a result of the study, it is essential to raise awareness that undetected neuropathy in SCI, regardless of its mechanism, is common and may be a modifiable risk factor for skin and vascular complications; therefore, electrophysiologic testing should be considered when indicated. It also increases the awareness that metabolic risk factors of polyneuropathy should be ruled out.

Given the growing importance of neurostimulation and neuromodulation therapies, clinicians should be aware of peripheral neuropathic conditions before applying functional electrical stimulation (FES) of the peroneal nerve to the lower extremities. Complete NCS of lower extremities should be performed before treatment, especially in high-risk patients: females, those with SCI longer than 10 years, cervical SCI, and a history of ischial pressure injury. Future research should include longitudinal cohort studies examining electrodiagnostic changes in SCI patients and exploring the mechanisms, predisposing factors, and precipitating causes of peripheral neuropathic conditions following SCI.

## Conclusions

In people with SCI, the prevalence of sural neuropathy is 24%. All of them are categorized into a type without evidence of compressive neuropathy. The significant associated factors of sural neuropathy were female, time since SCI longer than 10 years, cervical SCI, and history of pressure injury at the ischium. Due to limitations in the study design and data collection for detecting neuropathy and risk factors, further longitudinal studies are needed to understand the neurophysiological deterioration following SCI and to confirm these findings.

### Data Archiving

The datasets generated and/or analysed during the current study are available from the corresponding author on reasonable request.
